# Clinical significance of the number of retrieved lymph nodes in early gastric cancer with submucosal invasion

**DOI:** 10.1097/MD.0000000000031721

**Published:** 2022-11-18

**Authors:** Dae Hoon Kim, Hyo Yung Yun, Dong Hee Ryu, Hye Sook Han, Joung-Ho Han, Ki Bae Kim, Hanlim Choi, Taek-Gu Lee

**Affiliations:** a Department of Surgery, Chungbuk National University Hospital, Korea; b Internal Medicine, Chungbuk National University Hospital, Korea; c Department of Surgery, Chungbuk National University College of Medicine, Korea; d Internal Medicine, Chungbuk National University College of Medicine, Korea.

**Keywords:** early gastric cancer, lymph node metastasis, prognosis

## Abstract

The prognosis of early gastric cancer (EGC) with submucosal invasion is favorable; however, several cases of recurrence have been reported even after curative gastrectomy. This study aimed to investigate risk factors and evaluate the clinical significance of the number of retrieved lymph nodes (LNs) in EGC with submucosal invasion. We retrospectively analyzed the data of 443 patients with gastric cancer with submucosal invasion after curative gastrectomy for recurrent risk factors. Recurrence was observed in 22 of the 443 gastric cancer patients with submucosal invasion. In the univariate analysis, the risk factors for recurrence were the number of retrieved LNs ≤ 25 and node metastasis. In the multivariate analysis, retrieved LNs ≤ 25 (hazard ratio [HR] = 5.754, *P*-value = .001) and node metastasis (HR = 3.031, *P*-value = .029) were independent risk factors for recurrence after curative gastrectomy. Body mass index was related to retrieved LNs ≤ 25 in univariate and multivariate analyses (HR = .510, *P* = .002). The number of retrieved LNs and node metastases were independent risk factors for EGC with submucosal invasion. For EGC with submucosal invasion, retrieved LNs > 25 are necessary for appropriate diagnosis and treatment.

## 1. Introduction

Recently, the survival rate of gastric cancer has improved due to advancements in diagnosis and surgical treatment, particularly due to the increase in the diagnosis of asymptomatic early gastric cancer (EGC) through improvements in diagnostic technology.^[1,2]^ Consequently, the number of gastric cancer surgery survivors has increased.^[1,3]^ Therefore, many physicians are interested in improving quality of life through limited lymphadenectomy, endoscopic submucosal dissection, and sentinel lymph node navigation surgery. However, the basic principles of gastric cancer treatment are extensive dissection of the lymph nodes (LNs) and complete removal of the primary tumor in patients with resectable cancer.^[4–7]^ The prognostic risk factors for EGC include LN metastasis,^[8,9]^ tumor size,^[9]^ histologic type,^[10]^ lymphovascular invasion,^[10]^ and age.^[9]^ Lymph node metastasis is the most important prognostic risk factor for recurrence in early gastric cancer,^[8,9]^ but the prognostic risk factors for EGC other than LN metastasis remain debatable. Lymph node metastasis is an important prognostic factor in EGC. Therefore, gastrectomy with LN dissection is the primary treatment.^[11]^ Based on the AJCC 5th staging system, more than 15 retrieved LNs are required for accurate staging.^[12]^ Many studies have reported that the number of retrieved LNs is correlated with better prognosis.^[13–15]^ The number of resected LNs for each specimen was determined by the extent of lymphadenectomy and thoroughness of LN retrieval.^[16]^ Lymph node dissection is important because LN metastasis is a significant prognostic factor for early gastric cancer. The optimal number of retrieved LNs in gastric cancer has been shown to be greater than 15.^[12,17,18]^ The aim of our study was to evaluate the relationship between the number of retrieved LNs and prognosis in early gastric cancer with submucosal invasion and to analyze the factors affecting the number of retrieved LNs.

## 2. Materials and methods

Among the 1953 patients who were pathologically diagnosed with gastric cancer and underwent gastrectomy between January 1995 and December 2014, 443 who were diagnosed with submucosal invasion after gastrectomy were enrolled in this study. We excluded patients who underwent non-curative surgery, had a history of other cancers, or had remnant stomach cancer. Follow-up was conducted until October 2019, and the mean follow-up was 58.0 ± 40.9 months, with a median of 55.7 months. Recurrence patterns were classified into 4 categories: locoregional recurrence, peritoneal dissemination, hematogenous metastasis, and distant LNs. Gastric cancer surgery was performed according to the Japanese Gastric Cancer Treatment Guidelines.^[19]^ Pathological staging was conducted according to the AJCC 7th edition.^[20]^ The patients were followed up every 6 months after surgery for 5 years. The follow-up evaluation consisted of history taking, physical examination, laboratory findings, endoscopy, chest radiography, and computed tomography. Magnetic resonance imaging, positron emission tomography, and bone scans were performed when required. Recurrence was determined using a retrospective review of medical records.

We retrospectively analyzed the risk factors for recurrence in patients with submucosal invasion of the gastric cancer. All statistical analyses were performed using IBM SPSS software package (version 21.0; IBM Co., Armonk, NY, USA). The Kaplan–Meier method was used to analyze the risk factors for recurrence, and the log-rank test was used to analyze statistical significance. Chi-square tests were used to analyze clinicopathological correlations, and Cox proportional hazards models and logistic regression analyses were used for multivariate analyses. The relationship between retrieved LNs and metastatic LNs was analyzed using Spearman’s rank correlation coefficient. *A P*-value < .05 was considered at significant. This study was approved by our institutional review board.

## 3. Results

**Figure 1.**
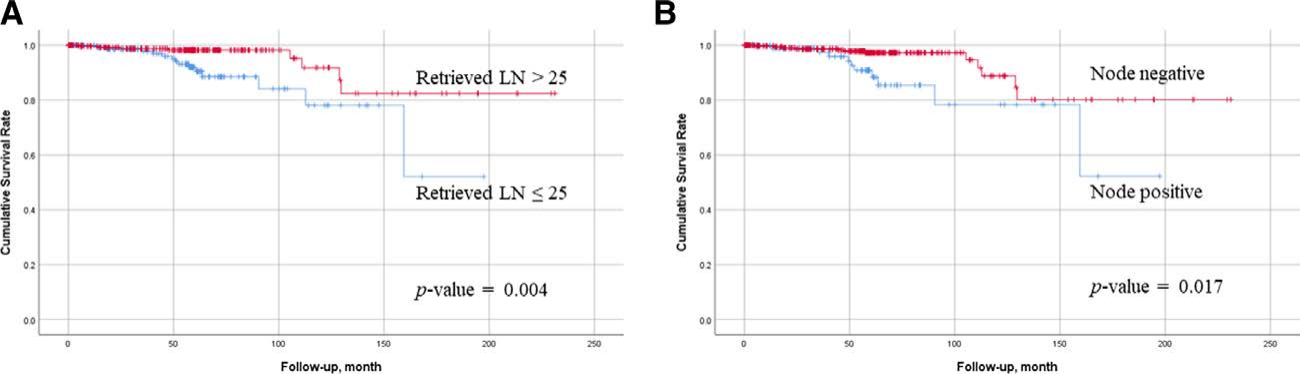
The recurrent graph according to the number of retrieved lymph nodes (A), and nodal status (B).

Among the 443 patients, 300 (67.7%) were male and 143 (32.3%) were female, with a mean age of 62.2 ± 10.6 years. Recurrence was observed in 22 of the 447 patients with submucosal gastric cancer. Complications occurred in 71 patients (16.0%), and the most common was wound complications (42 patients). Recurrence was observed at 47 sites in 22 patients, and the most common recurrence pattern was hematogenous (Table 1). The mean number of retrieved LNs was 31.0 ± 13.8. In the univariate analysis, retrieved LNs ≤ 25 (*P*-value = .004), complications (*P*-value = .002) and node metastasis (*P*-value = .017) were prognostic risk factors. The 5-years disease-specific survival (DSS) of retrieved LNs ≤ 25 and greater than 25 were 92.1% and 98.2%, respectively. According to node metastasis, the 5-years DSS rates for node negativity and positivity were 97.4% and 91.3%, respectively (Table 2, Fig. 1). In multivariate analysis, retrieved LNs ≤ 25 (hazard ratio [HR] = 5.754, *P*-value = .001) and node metastasis (HR = 3.031, *P*-value = .029) were independent prognostic risk factors (Table 3).

**Table 1:** Recurrent patterns of submucosal invasion in gastric cancer.

Peritoneal	Hematogenous	Locoregional	Distant LN	Total
10	15	10	7	42

**Table 2:** Univariate analysis of prognostic risk factors of submucosal invasion in gastric cancer.

Variables	N (%)	2 DSS	5 DSS	*P* value
Overall	443 (100.0%)	99.0%	95.9%	
Sex				.158
Male	300 (67.7%)	98.9%	94.9%	
Female	143 (32.3%)	99.1%	98.1%	
Age				.844
<60	163 (36.8%)	98.6%	96.0%	
≥60	280 (63.2%)	99.2%	95.8%	
Location of tumor				.867
Lower	256 (57.8%)	99.5%	96.3%	
Middle	153 (34.5%)	97.8%	95.2%	
Upper	34 (7.7%)	100.0%	95.5%	
Size				.062
<5 cm	382 (86.2%)	98.8%	96.9%	
≥5 cm	61 (13.8%)	100.0%	89.3%	
BMI				.077
<25 kg/m^2^	296 (66.2%)	98.8%	96.8%	
≥25 kg/m^2^	147 (32.9%)	99.3%	94.0%	
Retrieved LN				.004
≤25	273 (38.4%)	100.0%	92.1%	
>25	170 (61.6%)	99.2%	98.2%	
Complications				.002
Negative	372 (84.0%)	98.8%	95.2%	
Positive	71 (16.0%)	100.0%	100.0%	
Nodal status				.017
Negative	342 (77.2%)	99.7%	97.4%	
Positive	101 (22.8%)	96.7%	91.3%	
Differentiation				.441
Well differentiated	316 (71.3%)	99.3%	96.2%	
Poorly differentiated	127 (28.7%)	98.3%	95.3%	
Lymphovascular invasion				.187
Negative	339 (76.5%)	99.3%	96.7%	
Positive	104 (23.5%)	97.8%	93.2%	
Perineural invasion				.447
Negative	436 (98.4%)	98.9%	95.8%	
Positive	7 (1.6%)	100.0%	100.0%	

2 DSS = Two-year disease-specific survival, 5 DSS = Five-year disease-specific survival.

**Table 3:** Multivariate analysis of prognostic risk factors of submucosal invasion in gastric cancer.

Variables	*β*-coefficient	SE	95% CI	HR	*P* value
Sex					
Male					
Female	–1.002	0.579	0.118 ~ 1.138	0.366	.082
Age					
<60					
≥60	0.206	0.487	0.474 ~ 3.191	1.229	.671
Location of tumor					.411
Lower					
Middle	0.505	0.479	0.648 ~ 4.235	1.657	.292
Upper	0.888	0.812	0.494 ~ 11.949	2.461	.274
Size					
<5 cm					
≥5 cm	0.809	0.528	0.798 ~ 6.319	2.246	.125
BMI					
<25 kg/m^2^					
≥25 kg/m^2^	0.280	0.462	0.535 ~ 3.272	1.324	.544
Retrieved LN					
>25					
≤25	1.750	0.511	2.112 ~ 15.680	5.754	.001
Complications					
Negative					
Positive	0.083	0.665	0.295 ~ 3.998	1.086	.901
Nodal status					
Negative					
Positive	1.109	0.509	1.118 ~ 8.217	3.031	.029
Differentiation					
Well differentiated					
Poorly differentiated	0.532	0.495	0.646 ~ 4.488	1.702	.282
Lymphovascular invasion					
Negative					
Positive	0.444	0.558	0.523–4.651	0.1559	.426
Perineural invasion					
Negative					
Positive	0.490	1.166	0.166–16.041	1.633	.674

According to the number of retrieved LNs, 5-years DSS of retrieved LNs < 15, 16 to 20, 21 to 25, 26 to 30, and > 31 LNs were 93.2%, 89.7%, 93.4%, 100%, and 97.5%, respectively (*P*-value = .046) (Table 4).

**Table 4:** Five-year DSS according to number of retrieved LNs.

□LNs	N	2 DSS	5 DSS	*P* value
				.046
≤15	44	100.0%	93.2%	
16–20	56	98.0%	89.7%	
21–25	70	98.3%	93.4%	
26–30	75	100.0%	100.0%	
> 31	198	98.9%	97.5%	

**Figure 2.**
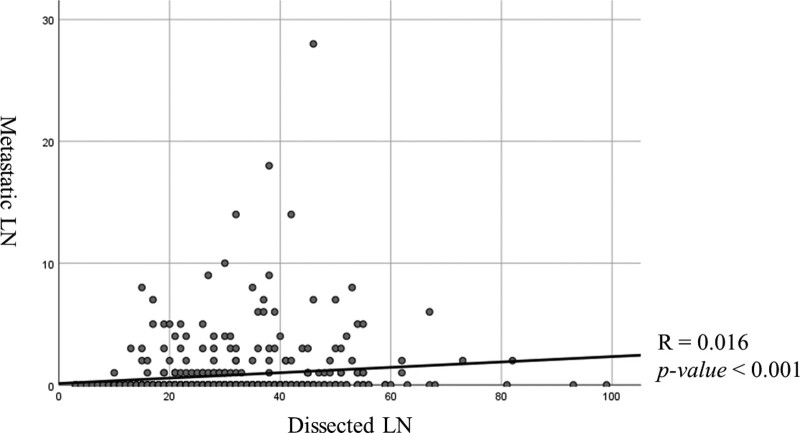
Correlation between the number of lymph nodes metastasis and retrieved lymph nodes. The relation between these was statistically significant. (*R* = 0.016, *P*-value < 0.001).

Age, sex, tumor location, tumor size, complications, differentiation, lymphovascular invasion, and perineural invasion were not associated with the number of retrieved lymph nodes. However, BMI (body mass index) and node metastasis were related with number of retrieved LNs fewer than 25 in univariate analysis. In multivariate analysis (Table 5), a BMI greater than 25 kg/m^2^ (HR = 0.510, *P*-value = .002) and node metastasis (HR = 2.084, *P*-value = .006) were independent prognostic factors of retrieved LNs ≤ 25 (Table 6). There was a statistically significant correlation between the number of retrieved and metastatic LNs (*r* = .167, *P* < .001) (Fig. 2).

**Table 5:** Univariate analysis of the risk factors of retrieved lymph nodes fewer than 20.

Variables	Number of retrieved LN		*P* value
	<25	≥25	
Sex			.308
Male	120 (40.0%)	180 (60.0%)	
Female	50 (35.0%)	93 (65.0%)	
Age			.357
<60	58 (35.6%)	105 (64.4%)	
≥60	112 (40.0%)	168 (60.0%)	
Location of tumor			.137
Lower	108 (42.2%)	148 (58.8%)	
Middle	52 (34.0%)	101 (66.0%)	
Upper	10 (29.4%)	24 (70.6%)	
Size			.495
<5cm	149 (39.0%)	233 (61.0%)	
≥5	21 (34.4%)	40 (65.6%)	
BMI (kg/m^2^)			.005
<25	100 (33.8%)	196 (66.2%)	
≥25	70 (47.6%)	77 (52.4%)	
Complications			.841
Negative	142 (38.2%)	230 (61.8%)	
Positive	28 (39.4%)	43 (60.6%)	
Nodal status			.006
Negative	143 (41.8%)	199 (58.2%)	
Positive	27 (26.7%)	74 (73.3%)	
Differentiation			.095
well differentiated	129 (40.8%)	187 (59.2%)	
poorly differentiated	41 (32.3%)	86 (67.7%)	
Lymphovascular invasion			.930
Negative	132 (38.5%)	211 (61.5%)	
Positive	38 (38.0%)	62 (62.0%)	
Perineural invasion			.186
Negative	169 (38.8%)	267 (61.2%)	
Positive	1 (14.3%)	6 (85.7%)	

**Table 6:** Multivariate analysis of the risk factors of retrieved lymph nodes fewer than 25.

Variables	*β*-coefficient	SE	95% CI	HR	*P* value
Sex					
Male					
Female	0.210	0.224	0.796–1.912	1.233	.348
Age					
<60					
≥60	–0.163	0.219	0.554–1.304	0.850	.456
Location of tumor					.185
Lower					
Middle	0.311	0.222	0.883–2.111	1.365	.161
Upper	0.586	0.408	0.808–3.994	1.797	.151
Size					
<5 cm					
≥5 cm	0.261	0.307	0.711–2.370	1.298	.395
BMI					
<25 kg/m^2^					
≥25 kg/m^2^	–0.674	0.214	0.335–0.775	0.510	.002
Complications					
Negative					
Positive	0.093	0.276	0.639–1.884	1.097	.737
Nodal status					
Negative					
Positive	0.735	0.270	1.229–3.536	2.084	.006
Differentiation					
Well differentiated					
Poorly differentiated	0.192	0.236	0.762–1.925	1.211	.418
Lymphovascular invasion					
Negative					
Positive	–0.159	0.257	0.516–1.411	0.853	.537
Perineural invasion					
Negative					
Positive	1.321	1.101	0.433–32.450	3.474	.230

## 4. Discussion

The prevalence of LN metastasis in EGC with submucosal invasion is reported to be 19.4% to 25.3%, and LN metastasis was observed in 22.3% of cases, similar to our results.^[2,10,18,21,22]^ LN metastasis in EGC with submucosal invasion is a common and a significant prognostic factor. The risk factors for LN metastasis in EGC are tumor size and lymphatic invasion.^[16,21]^ LN metastasis is a poor prognostic factor for EGC.^[8,9]^ The 5-years DSS rates for node negativity and node positivity were 97.4% and 91.3%, respectively. Our results also showed poor prognosis for EGC in the presence of LN metastasis. In addition, retrieved LNs < 25 was an independent prognostic risk factor for poor prognosis. Many studies have reported poor prognosis when the number of retrieved LNs was small.^[13,17,18,23–26]^ Although the number of appropriately retrieved LNs remains controversial, the 5th edition of AJCC requires at least 15 LN dissections for accurate staging.^[12]^ Roviello et al^[18]^ reported a good prognosis when more than 15 LNs were retrieved from EGC, even in node-negative patients. Lee et al^[17]^ reported significant differences in the number of metastatic LNs and survival in stage IIIA between retrieved LNs greater than 15 and < 15, and argued that at least 15 LNs should be examined for appropriate staging. Karphe et al^[25]^ reported similar results, in that survival significantly increased in stage II, IIIA, and IIIB patients when more than 15 LNs were examined. Haudahl et al^[27]^ also reported an improvement in the survival of patients with ≥ 15 LNs. Currently, many guidelines suggest that the cutoff number of retrieved LNs is ≥ 15.^[17,19,25–31]^ Haung et al^[23]^ reported that a number of examined LNs greater than 15 was associated with the detection of a larger number of positive LNs and presented with better long-term survival. In addition, they claimed that the minimal and optimal thresholds of the examined LNs that reduced stage migration and improved prognosis were 17 and 33 LNs, respectively. Zhao et al^[31]^ argued that > 15 retrieved LNs were not sufficient for advanced gastric cancer and that ≥ 25 LNs were needed for accurate staging. They reported that retrieval of 25 to 29 LNs could provide a better survival benefit than retrieval of < 25 or ≥ 30 LNs in advanced gastric cancer, and argued that retrieval of more than 25 LNs was necessary. Kim et al^[30]^ reported that the number of retrieved LNs was not related to prognosis in node-negative patients, but a higher number of retrieved LNs was associated with a better prognosis in node-positive patients. On this basis, he argued that retrieval of greater than 15 LNs was insufficient for curative gastrectomy, even for EGC. In our study, the 5-years DSS in univariate analysis was 92.1% when the number of retrieved LNs was less than 25 in submucosal invasion EGC and 98.2% when the number of retrieved LNs was > 25; this was a significant difference. In multivariate analysis, 25 or fewer retrieved LNs 25 (HR = 5.754, *P* = .001) was a significant risk factor for recurrence. In addition, our data showed that an increase in the number of examined LNs in the Spearman’s rank correlation coefficient analysis was associated with a higher number of positive LNs. Based on our data, there is a correlation between the number of resected LHs and the number of positive LNs, with resection of at least 25 LNs being appropriate for the treatment of EGC with submucosal invasion.

Examining fewer LNs leads to an underestimation of staging, which in turn leads to stage migration. This phenomenon is known as the Will-Roger phenomenon.^[32]^ Stage migration can affect prognosis, as explained by Haung et al.^[23]^ Among advanced-stage patients with fewer examined LNs, some could be misdiagnosed as having a lower stage, owing to insufficient sampling, resulting in lower survival. In addition, sampling a larger number of examined LNs can increase the likelihood of more accurate identification of more advanced N stages requiring adjuvant chemotherapy, allowing timely treatment that results in a good prognosis. Our data were similar to those of the aforementioned studies. In our study, the 5-years DSS of retrieved LNs less than 15, 16 to 20, 21 to 25, 26 to 30, and > 31 were 93.2%, 89.7%, 96.4%, 100%, and 97.5%, respectively. Our data showed that the prognosis of EGC with number of retrieved LNs 26 to 30 was better than that of the other groups. Even when the number of retrieved LNs was limited, it remained a significant independent prognostic factor in multivariate analysis. In addition, there was a significant correlation between the number of retrieved LNs with number of metastatic LNs. This implies that staging would be incorrectly lower with fewer retrieved LNs. Although our data have limitations of coming from a single institution and a small number of cases, they suggest retrieval of more than 25 LNs for the appropriate treatment of EGCs.

The number of retrieved LNs is affected by the extent of lymphadenectomy,^[33–35]^ techniques used for the retrieval of LNs,^[36,37]^ histological examination,^[38]^ and BMI.^[39–41]^ Because our data were limited to a single institution and submucosal invasion of EGCs, they might demonstrate a lower impact on the extent of lymphadenectomy, techniques for retrieval of LNs, and histologic examination. Chen et al^[39]^ reported that 26.3% of obese patients with BMI ≥ 25 kg/m2 had fewer than 15 retrieved LNs. Feng et al^[41]^ also reported that the number of retrieved LNs was low in patients with obesity. Based on clinical experience, obesity is associated with thickness of the abdominal wall and massive adipose tissue in the abdominal cavity, complicating surgical resection.^[42]^ Moreover, LNs are located in the deep adipose tissue around major vessels, hindering resection in obese patients.^[43]^ In our study, although obesity was not associated with DSS, 70 of 144 obese patients (47.6%) had fewer than 25 retrieved LNs, which was significantly higher than the proportion of patients with a BMI < 25 kg/m2 (100 of 296 patients, 33.8%). In multivariate analysis, obesity was an independent risk factor for the number of retrieved LNs.

In a randomized clinical trial, it is well known that the extent of node dissection performed by an experienced surgeon does not affect complications after gastrectomy.^[44]^ Sun et al^[45]^ reported no association between the number of retrieved lymph nodes and complications in patients with gastric cancer. In our study, there was no association between the number of retrieved LNs and number of complications.

Our study has limitations in that it investigated a single institution’s experience with a small sample size, which might have resulted in a bias during the analysis. Therefore, a multicenter study with a larger sample size is needed to confirm our results. Node metastasis and fewer than 25 retrieved LNs were associated with poor prognosis in EGC with submucosal invasion, and the number of retrieved LNs was small in obese patients. Extended lymphadenectomy is necessary even for early gastric cancer, and more careful LN dissection is required in obese patients.

## Author contributions

**Conceptualization:** H.Y Yun, D. H. Kim.

**Data curation:** D. H. Kim.

**Investigation:** D. H. Kim, H. Choi.

**Methodology:** H. Y. Yun.

**Supervision:** H. Y. Yun.

**Project administration:** T G. Lee, J H Han, H S Han.

**Writing—original draft:** D. H. Kim.

**Writing—review & editing:** Y. H. Yun, D. H. Yun.
